# Assessing the association between genetic and phenotypic features of dilated cardiomyopathy and outcome in patients with coronary artery disease

**DOI:** 10.1002/ejhf.3033

**Published:** 2023-10-05

**Authors:** Richard E. Jones, Daniel J. Hammersley, Sean Zheng, Kathryn A. McGurk, Antonio de Marvao, Pantazis I. Theotokis, Ruth Owen, Upasana Tayal, Gillian Rea, Suzan Hatipoglu, Rachel J. Buchan, Lukas Mach, Lara Curran, Amrit S. Lota, François Simard, Rohin K. Reddy, Suprateeka Talukder, Won Young Yoon, Ali Vazir, Dudley J. Pennell, Declan P. O'Regan, A. John Baksi, Brian P. Halliday, James S. Ware, Sanjay K. Prasad

**Affiliations:** ^1^ National Heart and Lung Institute Imperial College London London UK; ^2^ Royal Brompton and Harefield Hospitals Guy's and St Thomas' NHS Foundation Trust London UK; ^3^ Anglia Ruskin University Chelmsford UK; ^4^ Essex Cardiothoracic Centre Basildon UK; ^5^ MRC London Institute of Medical Sciences Imperial College London London UK; ^6^ Department of Women and Children's Health King's College London London UK; ^7^ British Heart Foundation Centre of Research Excellence School of Cardiovascular Medicine and Sciences, King's College London London UK; ^8^ Department of Medical Statistics London School of Hygiene and Tropical Medicine London UK

**Keywords:** Coronary artery disease, Dilated cardiomyopathy, Rare pathogenic genetic variants, Cardiovascular magnetic resonance

## Abstract

**Aims:**

To examine the relevance of genetic and cardiovascular magnetic resonance (CMR) features of dilated cardiomyopathy (DCM) in individuals with coronary artery disease (CAD).

**Methods and results:**

This study includes two cohorts. First, individuals with CAD recruited into the UK Biobank (UKB) were evaluated. Second, patients with CAD referred to a tertiary centre for evaluation with late gadolinium enhancement (LGE)‐CMR were recruited (London cohort); patients underwent genetic sequencing as part of the research protocol and long‐term follow‐up. From 31 154 individuals with CAD recruited to UKB, rare pathogenic variants in DCM genes were associated with increased risk of death or major adverse cardiac events (hazard ratio 1.57, 95% confidence interval [CI] 1.22–2.01, *p* < 0.001). Of 1619 individuals with CAD included from the UKB CMR substudy, participants with a rare variant in a DCM‐associated gene had lower left ventricular ejection fraction (LVEF) compared to genotype negative individuals (mean 47 ± 10% vs. 57 ± 8%, *p* < 0.001). Of 453 patients in the London cohort, 63 (14%) had non‐infarct pattern LGE (NI‐LGE) on CMR. Patients with NI‐LGE had lower LVEF (mean 38 ± 18% vs. 48 ± 16%, *p* < 0.001) compared to patients without NI‐LGE, with no significant difference in the burden of rare protein altering variants in DCM‐associated genes between groups (9.5% vs. 6.7%, odds ratio 1.5, 95% CI 0.4–4.3, *p* = 0.4). NI‐LGE was not independently associated with adverse clinical outcomes.

**Conclusion:**

Rare pathogenic variants in DCM‐associated genes impact left ventricular remodelling and outcomes in stable CAD. NI‐LGE is associated with adverse remodelling but is not an independent predictor of outcome and had no rare genetic basis in our study.

## Graphical abstract

In this study, two independent cohorts were used to investigate for cardiovascular magnetic resonance (CMR) and genetic features of dilated cardiomyopathy (DCM) in individuals with coronary artery disease (CAD). First, the UK Biobank was used to assess for the association between rare variants in DCM‐associated genes, phenotype and outcomes in participants with CAD. Individuals with rare pathogenic variants in DCM‐associated genes had greater adverse cardiac remodelling and worse clinical outcomes as compared to genotype negative individuals; a result that suggests the presence of a high‐risk subgroup of patients with CAD and concomitant vulnerability to DCM. Second, the prevalence, distribution and prognostic relevance of non‐infarct pattern late gadolinium enhancement (NI‐LGE) in patients recruited into a CMR and genetics registry was assessed (the London cohort). NI‐LGE was associated with adverse left ventricular remodelling but was not an independent prognostic indicator. There was no enrichment of rare variants in DCM‐associated genes in patients with NI‐LGE compared to patients without this imaging biomarker. LAVi, indexed left atrial volume; LVEDVi, indexed left ventricular end‐diastolic volume; LVEF, left ventricular ejection fraction; MI, myocardial infarction; RVEF, right ventricular ejection fraction; TTNtv, titin truncating variant.

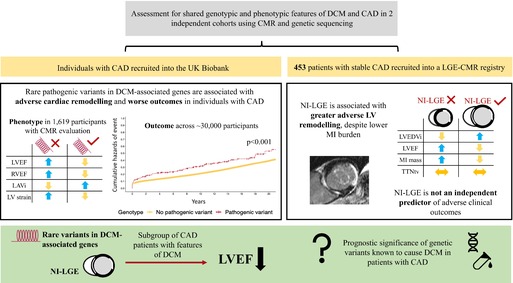

## Introduction

Cardiovascular magnetic resonance (CMR) and genetic sequencing offer precision disease characterization and risk stratification in patients with dilated cardiomyopathy (DCM).^1^, ^2^ The interaction between environmental modifiers (including alcohol excess, pregnancy, chemotherapy and myocarditis) with underlying genetic susceptibility is increasingly recognized in patients with DCM.^1^, ^2^, ^3^, ^4^ Whilst the utility of CMR in ischaemic heart disease is established, the role of genetic sequencing is largely unknown. A large study found that approximately 60% of patients with heart failure (HF) and a pathogenic or likely pathogenic cardiomyopathy variant had ischaemic heart disease, demonstrating coexistence of two distinct potential causes of cardiac dysfunction in a subgroup of patients.^5^ Whether rare variants in DCM‐associated genes affect phenotype and outcomes in patients with coronary artery disease (CAD) requires further clarification. Prior reports of phenotypic overlap between these two diseases include non‐ischaemic scar patterns in patients with a primary diagnosis of CAD (online supplementary *Figure* *S2*)^6^, ^7^ and extensive heterogeneity in the degree of adverse remodelling for equivalent levels of infarct scar in patients with stable CAD. In clinical practice, these patients may be considered to have ‘dual pathology’ of coronary heart disease with intercurrent intrinsic non‐ischaemic myocardial disease. This subjective diagnostic label can alter downstream management decisions, including suitability for coronary revascularization and recommendations for family screening and genetic profiling.

In this study, we seek to use data from a deeply characterized patient cohort, alongside orthogonal evidence from a large population‐based registry, to determine the prevalence and prognostic significance of key DCM markers (genetic and phenotypic) in individuals with stable CAD and to understand their impact on adverse cardiac remodelling.

## Methods

### UK Biobank cohort

The UK Biobank (UKB) is a nationwide biomedical cohort study.^8^, ^9^ The study was reviewed by the National Research Ethics Service (11/NW/0382, 21/NW/0157) and written informed consent was required from all participants. The study adheres to the principles set out in the Declaration of Helsinki and the data were de‐identified. We curated two groups: the first group comprised individuals with CAD and both genetic and CMR evaluation (UKB Group 1). This group permitted the assessment of cardiac phenotype in individuals with CAD who harbour rare pathogenic variants in DCM‐associated genes. The second group (UKB Group 2) comprised all individuals with CAD who underwent genetic evaluation (i.e., beyond those recruited into the CMR substudy). This group was used to assess clinical outcomes in individuals with CAD and carriers of rare pathogenic variants in DCM‐associated genes. The UKB fields used to determine the diagnosis of CAD are detailed in online supplementary *Table* *S1*.

From the UKB whole exome sequencing data, carriers of rare variants (minor allele frequency <0.1% and filtering allele frequency <0.00004 in gnomAD) with appropriate disease‐causing mechanisms in 12 definitive or strong evidence DCM genes (*BAG3*, *DES*, *DSP*, *FLNC*, *LMNA*, *MYH7*, *PLN*, *RBM20*, *SCN5A*, *TNNC1*, *TNNT2*, and *TTN*) were identified. For *TTN*, only cardiac expressed exons with PSI >0.9 were included.^10^ Variants were then filtered to identify those that would be considered pathogenic or likely pathogenic in DCM (excluding variants with evidence for pathogenicity in hypertrophic cardiomyopathy), using CardioClassifier^11^ and ClinVar. UKB whole‐exome sequencing data were processed using VEP (version 105^12^) with plugins for Genome Aggregation database (gnomAD),^13^ LOFTEE^13^ and SpliceAI.^14^

Participants recruited in the imaging substudy underwent CMR at 1.5 T. Segmentation of the cine imaging was undertaken using a deep learning neural network with subsequent calculation of biventricular volumes, left atrial volume and strain indices as previously described.^15^

UKB mapped: (i) primary care data; (ii) International Classification of Diseases (ICD)‐9 and ICD‐10 codes from hospital inpatient data; (iii) ICD‐10 codes from Death Register records and; (iv) self‐reported medical conditions to ICD‐10 codes. The earliest occurrence of each event in a participant's lifetime was reported. The primary endpoint was a composite of death or major adverse cardiovascular events (MACE), the latter defined as a diagnostic code for cardiac arrest or HF. Secondary endpoints included the individual components of the primary endpoint and atrial fibrillation. See online supplementary material for extended methods.

### London cohort

Patients with stable CAD undergoing CMR were prospectively recruited into a registry from 2009 to 2016. The study conformed with the principles outlined in the Declaration of Helsinki. A CMR scan at 1.5 T (Siemens Sonata/Avanto) was either undertaken on the day of recruitment or, in a minority of patients, had been performed at an earlier date within the institution. Consenting patients underwent biobanking of whole blood for genetic analysis.

Coronary artery disease was confirmed by either the presence of: (i) severe epicardial CAD; (ii) previous coronary revascularization, or (iii) history of prior myocardial infarction verified on CMR. Severe epicardial CAD was defined as ≥75% stenosis in the left main stem or proximal left anterior descending artery, or ≥75% in any other two epicardial coronary arteries.^16^ The exclusion criteria were myocardial infarction within 40 days prior to CMR, severe primary valve disease (or prior valvular intervention) or a confirmed primary diagnosis of a non‐ischaemic cardiomyopathic process (e.g. myocarditis, sarcoidosis, or dilated, hypertrophic or infiltrative cardiomyopathy).

The presence and distribution of non‐infarct pattern late gadolinium enhancement (NI‐LGE) was confirmed by two independent Level 3 accredited CMR operators blinded to the clinical and outcome data. DNA extraction was performed on available whole blood using automated platforms followed by targeted sequencing on the Solid 5500×l or Illumina NextSeq platforms. Samples from both platforms were jointly analysed, annotated and filtered using a customized bioinformatics pipeline. Additionally, sequencing data from a healthy volunteer cohort and the reference population, gnomAD, were used.^13^ Analysis was performed on genes robustly associated with DCM. Protein altering variants (PAVs) in 11 DCM genes were assessed (*BAG3*, *DSP*, *DES*, *LMNA*, *MYH7*, *PLN*, *RBM20*, *SCN5A*, *TNNC1*, *TNNT2* and *TTN*). We restricted analyses to variant classes known to cause disease and implicated in mechanisms of pathogenesis (online supplementary *Table* *S2*); extended analysis using CardioClassifier^11^ as an additional annotation step was also performed. *TTN* truncating variants (tv) in exons with a cardiac percentage spliced in (PSI) >90%^10^ were included. Data on *FLNC* were not available as a proportion of patients were sequenced prior to filamin C variants being recognized as a cause of DCM. Follow‐up events were adjudicated by an independent panel of cardiologists. The primary outcome was a composite of death or MACE. MACE was defined as life threatening arrhythmia (sudden cardiac death, appropriate implantable cardioverter‐defibrillator shock for a ventricular tachyarrhythmia, successful resuscitation following ventricular fibrillation or haemodynamically unstable ventricular tachycardia) or HF event (HF hospitalization, HF death, cardiac transplantation or left ventricular assist device insertion). See online supplementary material for extended methods.

### Statistical analysis

Baseline characteristics were summarized as frequency (%) for categorical variables and mean (standard deviation [SD]) or median (interquartile range [IQR]) for continuous variables.

In the UKB, comparison of CMR traits in CAD participants with and without a rare variant in a DCM‐associated gene was performed using analysis of covariance, adjusting for age, age^2^, sex, systolic blood pressure and for the non‐indexed traits, body surface area. Clinical outcomes were analysed in participants with CAD, stratified by presence or absence of a rare variant in a DCM‐associated gene. Cox proportional hazards were calculated for lifetime risk and incident risk of clinical events, adjusting for age, age^2^, sex, and genetic principal components 1–10. For the non‐fatal secondary outcomes, competing risk analysis was performed using the cause‐specific survival method. Time to event was censored at first event for each outcome, death, or last recorded follow‐up. Individuals with events preceding CAD diagnosis were excluded from the incident outcome analysis.

In the London cohort, Kaplan–Meier curves were fitted to describe the cumulative incidence of the primary outcome stratified by presence or absence of NI‐LGE and were compared using the log‐rank test. Cox regression analyses were performed to explore the association between NI‐LGE presence and distribution and the primary outcome. The multivariable analyses were adjusted for age, sex and LVEF (model A) and a subset of covariables from *Table* *1* (model B). To select the covariables in model B, a forward stepwise procedure was applied with *p* > 0.10 as the criterion for exclusion. The sensitivity analyses are detailed in online supplementary Methods. In the primary genetic analysis, we tested whether CAD patients with NI‐LGE had a higher mutation burden in rare PAVs (including a separate analysis for *TTN*tv) compared to (i) CAD patients without NI‐LGE, (ii) healthy volunteers (HVOLs), and (iii) unrelated individuals from gnomAD.

**Table 1:** Imaging traits in individuals with coronary artery disease recruited into the UK Biobank Group 1

Variable	No rare variant (*n* = 1611)	With rare variant (*n* = 8)	*p*‐value
LV ejection fraction (%)	56.7 (7.9)	46.7 (10.3)	<0.001
LVEDVi (ml/m^2^)	83.9 (17.7)	86.8 (23.8)	0.75
LVESVi (ml/m^2^)	36.9 (13.5)	47.3 (18.3)	0.03
LVSVi (ml/m^2^)	47.0 (9.0)	39.5 (9.2)	0.01
LV mass (g)	99.5 (22.9)	111.0 (30.2)	0.19
LAVi (ml/m^2^)	42.5 (14.2)	52.2 (28.7)	0.11
LA ejection fraction (%)	56.5 (11.3)	45.4 (18.4)	0.02
RV ejection fraction (%)	55.8 (6.9)	49.8 (8.7)	0.01
RVEDVi (ml/m^2^)	85.2 (15.6)	76.2 (17.7)	0.05
RVESVi (ml/m^2^)	37.7 (9.5)	38.4 (11.3)	0.99
RVSVi (ml/m^2^)	47.5 (9.7)	37.8 (11.1)	0.003
RAVi (ml/m^2^)	47.2 (15.5)	50.6 (21.9)	0.72
RA ejection fraction (%)	43.7 (9.8)	34.8 (12.5)	0.03
LV global radial strain (%)	42.1 (9.3)	31.0 (9.7)	<0.001
LV global circumferential strain (%)	−20.7 (4.2)	−15.1 (4.4)	<0.001
LV global longitudinal strain (%)	−17.7 (3.3)	−13.9 (4.1)	0.005

Values are mean (standard deviation). Analyses were adjusted for age, sex, systolic blood pressure and for non‐indexed values, body surface area, using analysis of covariance test.

LA, left atrial; LAVi, indexed left atrial volume; LV, left ventricular; LVEDVi, indexed left ventricular end‐diastolic volume; LVESVi, indexed left ventricular end‐systolic volume; LVSVi, indexed left ventricular stroke volume; RA, right atrial; RAVi, indexed right atrial volume; RV, right ventricular; RVEDVi, indexed right ventricular end‐diastolic volume; RVESVi, indexed right ventricular end‐systolic volume; RVSVi, indexed right ventricular stroke volume.

## Results

### The UK Biobank cohort

#### Cardiac phenotype of individuals with coronary artery disease and carriers of rare pathogenic variants in dilated cardiomyopathy‐associated genes

The UKB was initially used to investigate the association between rare pathogenic variants in DCM‐associated genes and cardiac imaging traits in individuals with known CAD (UKB Group 1). In the 1619 participants with CAD who underwent CMR and whole‐exome sequencing, the presence of a pathogenic rare variant (*n* = 8 patients, 7 with *TTN*tvs) was associated with reduced biventricular ejection fraction (LVEF: 46.7 ± 10.3% vs. 56.7 ± 7.9%, *p* = 0.0003; right ventricular ejection fraction: 49.8 ± 8.7% vs. 55.8 ± 6.9%, *p* = 0.01), reduced indexed stroke volumes (left ventricular stroke volume: 39.5 ± 9.2 ml/m^2^ vs. 47.0 ± 9.0 ml/m^2^, *p* = 0.01; right ventricular stroke volume: 37.8 ± 11.1 ml/m^2^ vs. 47.5 ± 9.7 ml/m^2^, *p* = 0.003) and increased indexed left ventricular end‐systolic volume (47.3 ± 18.3 mL/m^2^ vs. 36.9 ± 13.5 ml/m^2^, *p* = 0.03 compared to individuals without a rare pathogenic variant (*n* = 1611) (*Table* *1*). Additionally, rare variant carrier status was associated with decreased left ventricular global strain patterns (radial: 31 ± 10% vs. 42 ± 9%, *p* < 0.001; circumferential: −15 ± 4% vs. −21 ± 4%, *p* < 0.001; longitudinal: −14 ± 4% vs. −18 ± 3%, *p* = 0.005) (*Table* *1*).

#### Clinical outcomes in individuals with coronary artery disease and carriers of rare pathogenic variants in dilated cardiomyopathy‐associated genes

**Figure 1:**
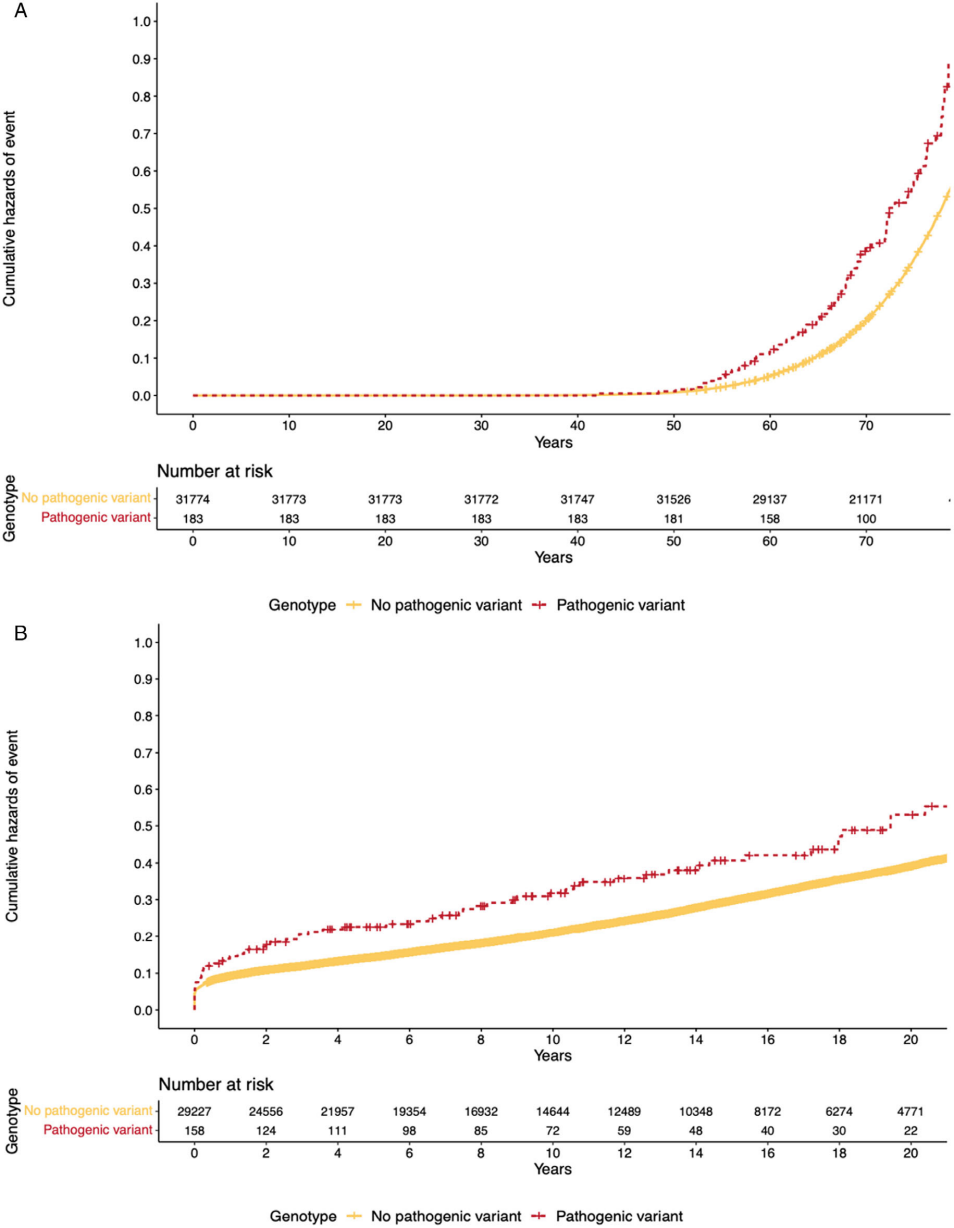
Clinical outcomes of UK Biobank participants with coronary artery disease stratified by presence or absence of rare pathogenic variants in dilated cardiomyopathy‐associated genes. (*A*) Cumulative hazard curves for lifetime risk of the primary endpoint in UK Biobank individuals with coronary artery disease, stratified by genotype; *p* < 0.001. (*B*) Cumulative hazard curves for incident risk of the primary outcome in UK Biobank individuals with coronary artery disease, stratified by genotype; *p* < 0.001. Incident outcome analysis includes participants with a known coronary artery disease diagnosis date, excluding individuals with a preceding event. All models adjusted for age, age^2^, sex, and principal components 1‐10.

The UKB was subsequently used to assess clinical outcomes in individuals with CAD and rare pathogenic variants in DCM‐associated genes (UKB Group 2); this cohort included 31 957 participants. Baseline demographics are presented in online supplementary *Tables* *S3* and *S4*. In this group, the presence of a pathogenic rare variant (*n* = 183, *n* = 130 individuals with *TTN*tv) was associated with increased lifetime risk of the primary endpoint (hazard ratio [HR] 1.67, 95% confidence interval [CI] 1.36–2.07, *p* < 0.001, *Figure* *1A*), HF (HR 1.94, 95% CI 1.52–2.48, *p* < 0.001) and atrial fibrillation (HR 2.18, 95% CI 1.74–2.74, *p* < 0.001), but not with survival (HR 1.19, 95% CI 0.87–1.61, *p* = 0.3). There were 31 154 participants with a known CAD diagnosis date. After a mean of 11.3 years follow‐up (SD 8.9 years), the presence of a pathogenic rare variant (*n* = 158 patients) was associated with time to the primary endpoint after diagnosis (HR 1.57, 95% CI 1.22–2.01, *p* < 0.001) (*Figure* *1B*), HF (HR 1.82, 95% CI 1.35–2.45, *p* < 0.001) and atrial fibrillation (HR 1.95, 95% CI 1.46–2.61, *p* < 0.001). There was no association with survival (HR 1.19, 95% CI 0.85 to 1.66, *p* = 0.3). Sensitivity analysis restricting only to carriers of *TTN*tvs showed similar associations with outcomes (online supplementary *Figure* *S1*).

### The London cohort

From 734 patients with suspected CAD, 453 were included in the final London cohort (*Table* *2* and online supplementary *Figure* *S2*). The mean age was 64 ± 10 years and mean LVEF 47 ± 17%. The final cohort included 426 (94%) patients with severe native CAD or prior coronary revascularization and 27 (6%) patients without documented CAD but a clinical history of myocardial infarction with ischaemic pattern LGE on CMR. DNA sequencing was performed on 273/453 (60%) patients on research grounds. The median follow‐up was 6.4 years (IQR: 5.1–7.9 years). NI‐LGE was confirmed in 63 (14%) patients (examples shown in online supplementary *Figure* *S3*).

**Table 2:** Baseline characteristics of the London cohort

Variable	Non‐infarct LGE		*p*‐value
	No (*n* = 390)	Yes (*n* = 63)	
**Demographics**			
Age (years)	64.1 (9.9)	66.8 (9.5)	0.04
Female sex	62 (15.9)	1 (1.6)	0.002
Caucasian	322 (82.6)	50 (79.4)	0.54
BMI (kg/m^2^)	27.9 (5.1)	27.5 (4.2)	0.63
Significant CAD^a^	366 (93.8)	60 (95.2)	0.95
CAD type			0.46
Single vessel	118 (32.2)	17 (28.3)	
2 vessels	108 (29.5)	15 (25.0)	
3 vessels	140 (38.3)	28 (46.7)	
Prior MI	295 (75.6)	35 (55.6)	<0.001
Hypertension	204 (52.3)	35 (55.6)	0.63
Diabetes mellitus	112 (28.7)	18 (28.6)	0.98
Prior PCI	202 (51.8)	26 (41.3)	0.12
Prior CABG	108 (27.7)	18 (28.6)	0.89
NYHA class			0.09
I	140 (36.1)	14 (22.2)	
II	167 (43.0)	32 (50.8)	
III or IV	81 (20.9)	17 (27.0)	
Baseline atrial fibrillation	66 (16.9)	10 (15.9)	0.84
**Medication history**			
Diuretic	174 (44.6)	36 (57.1)	0.06
Beta‐blocker	308 (79.0)	44 (69.8)	0.11
ACEi/ARB	332 (85.1)	49 (77.8)	0.14
Lipid‐lowering drug	344 (88.2)	56 (88.9)	0.88
Aldosterone antagonist	90 (23.1)	21 (33.3)	0.08
**CMR volumetric measurements**			
LVEF (%)	48.3 (16.0)	38.4 (18.2)	<0.001
LV mass indexed (g/m^2^)	76.7 (22.5)	93.6 (30.3)	<0.001
LVEDVi (ml/m^2^)	103.2 (38.2)	131.2 (48.2)	<0.001
RVEF (%)	59.1 (11.9)	51.9 (15.3)	<0.001
RVEDVi (ml/m^2^), median (IQR)	72.6 (61.9–85.4)	78.0 (68.0–89.8)	0.01
**CMR LGE characteristics**			
Any LGE present	349 (89.5)	63 (100.0)	0.007
Total LGE mass (g), median (IQR)	19.2 (7.8–31.7)	17.8 (8.9–31.6)	0.83
Infarct pattern LGE present	349 (89.5)	45 (71.4)	<0.001
Infarct pattern LGE mass (g), median (IQR)	19.2 (7.8–31.7)	11.5 (0.0–23.3)	0.001
No. infarcted segments, median (IQR)	5.0 (3.0–8.0)	3.0 (0.0–6.0)	<0.001
Predominant territory of infarct‐pattern LGE			0.13
Anterior	162 (46.4)	18 (40.0)	
Lateral	46 (13.2)	11 (24.4)	
Inferior	141 (40.4)	16 (35.6)	
NI‐LGE location			
Septal	0 (.)	37 (58.7)	
LV free‐wall	0 (.)	8 (12.7)	
Both	0 (.)	18 (28.6)	
NI‐LGE pattern			
Linear midwall	0 (.)	46 (73.0)	
Sub‐epicardial	0 (.)	5 (7.9)	
Multiple patterns	0 (.)	12 (19.0)	
Non‐infarct pattern LGE mass (g), median (IQR)	0.0 (0.0–0.0)	5.2 (2.7–9.2)	<0.001
DNA sequencing^b^	*n* = 210 sequenced	*n* = 63 sequenced	
Titin truncating variant	2 (1.0)	2 (3.2)	0.22
Rare protein altering variant	14 (6.7)	6 (9.5)	0.42

Continuous variables are reported as mean (standard deviation), or median (IQR). Categorical variables are reported as *n* (%). Continuous variables were compared with Student's *t*‐test or Mann–Whitney U test based on normality of data. Categorical variables were compared with *χ* ^2^ test or the Fisher exact test where applicable.

ACEi, angiotensin‐converting enzyme inhibitor; ARB, angiotensin II receptor blocker; BMI, body mass index; CABG, coronary artery bypass grafting; CAD, coronary artery disease; CMR, cardiovascular magnetic resonance; IQR, interquartile range; LGE, late gadolinium enhancement; LVEDVi, indexed left ventricular end‐diastolic volume; LVEF, left ventricular ejection fraction; MI, myocardial infarction; NI‐LGE, non‐infarct pattern late gadolinium enhancement; NYHA, New York Heart Association; PCI, percutaneous coronary intervention; RVEDVi, indexed right ventricular end‐systolic volume; RVEF, right ventricular ejection fraction.

^a^ Patients with evidence of severe CAD or a history of prior coronary revascularization.

^b^ Genetic sequencing was performed on 273/453 patients within the London cohort; 234 cases using the Solid platform and 39 cases using the Illumina platform.

#### Cardiac phenotype of coronary artery disease patients with rare protein altering variants in dilated cardiomyopathy‐associated genes

Across the London cohort, rare PAVs in DCM‐associated genes were identified in 20 patients; *TTN*tvs were detected in 4 patients (online supplementary *Table* *S5* for all rare variants and protein consequences identified in the London cohort). There was no significant enrichment of PAVs in patients with NI‐LGE as compared to patients without NI‐LGE (9.5% vs. 6.7%; odds ratio [OR] 1.5, 95% CI 0.4–4.3, *p* = 0.4), HVOLs (9.5% vs. 9.4%; OR 1.0, 95% CI 0.4–2.4, *p* = 1) or the reference population gnomAD (6.3% vs. 4.0%; OR 1.6, 95% CI 0.7–3.3, *p* = 0.2). Specifically evaluating *TTN*, there was not a significantly increased burden of *TTN*tv in patients with NI‐LGE as compared to patients without NI‐LGE (3.2% vs. 1.0%; OR 3.4, 95% CI 0.2–48.0, *p* = 0.2), HVOLs (3.2% vs. 0.7%; OR 4.6, 95% CI 0.5–22.0, *p* = 0.09) or gnomAD (1.6% vs. 0.3%; OR 5.3, 95% CI 0.6–20.0, *p* = 0.06). The mean LVEF of patients with and without a *TTN*tv was 33 ± 15% vs. 41 ± 16% respectively (*p* = 0.37). The baseline demographics of patients in the London cohort, stratified by *TTN*tv, are summarized in online supplementary *Table* *S6*. Extended genetic analysis is detailed in online supplementary Results and *Figure* *S4*.

#### Cardiac phenotype and outcomes of coronary artery disease patients with non‐infarct pattern late gadolinium enhancement on cardiovascular magnetic resonance

Patients with NI‐LGE had increased indexed left ventricular end‐diastolic volumes (131 ± 48 ml/m^2^ vs. 103 ± 38 ml/m^2^, *p* < 0.001) and a lower LVEF (38 ± 18% vs. 48 ± 16%, *p* < 0.001) compared to patients without NI‐LGE, despite a similar number of severely diseased coronary vessels (*p* = 0.46), decreased prevalence of prior clinical myocardial infarction (56% vs. 76%, *p* < 0.001) and reduced infarct LGE mass (median [IQR]:12 g [0–23 g] vs. 19 g [8–32 g], *p* = 0.001) (*Table* *2*). Sensitivity analyses exploring the association between cardiac phenotype and presence of NI‐LGE are detailed in online supplementary *Tables* *S7*–*S10*.

**Figure 2:**
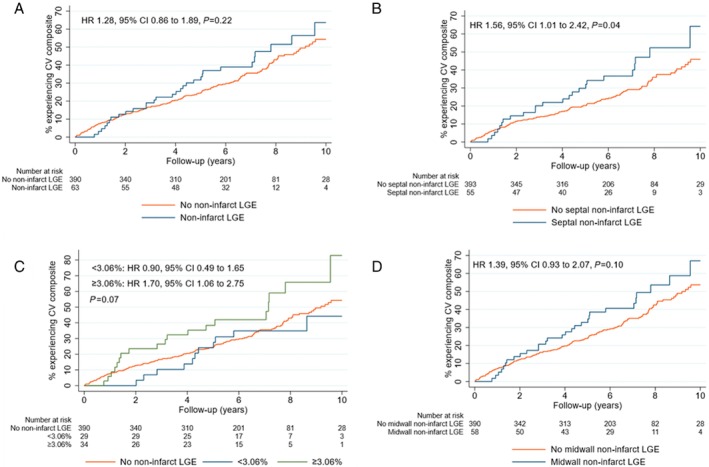
Kaplan–Meir plots for the primary composite endpoint of all‐cause mortality, major heart failure event and life‐threatening arrhythmia in the London cohort. (*A*) Cumulative incidence plots for the primary endpoint, stratified by presence or absence of non‐infarct pattern late gadolinium enhancement (LGE). The plot demonstrates that patients with non‐infarct pattern LGE did not have an increased cumulative incidence of the primary endpoint. (*B*) Cumulative incidence plots for the primary endpoint, stratified by presence or absence of non‐infarct pattern LGE within the ventricular septum. The plot demonstrates that patients with septal non‐infarct pattern LGE had an increased cumulative incidence of the primary endpoint. (*C*) Cumulative incidence plots for the primary endpoint, stratified by non‐infarct pattern LGE extent. The plot demonstrates that patients with non‐infarct pattern LGE mass above median did not have an increased cumulative incidence of the primary endpoint. (*D*) Cumulative incidence plots for the primary endpoint, stratified by presence or absence of mid‐wall non‐infarct pattern LGE. The plot demonstrates that patients with mid‐wall non‐infarct pattern LGE did not have an increased cumulative incidence of the primary endpoint. CI, confidence interval; CV, cardiovascular; HR, hazard ratio.

Over a median follow up of 6.4 years, 181 (40%) patients met the primary endpoint of all‐cause mortality, life‐threatening arrhythmia or major HF event. In patients with CAD, the presence of NI‐LGE was not associated with adverse events on univariate or multivariate analysis (*Figure* *2A* and online supplementary *Table* *S11*). The association between clinical outcomes and the location, pattern and extent of NI‐LGE was also assessed. Overall, 55 (12%) patients had NI‐LGE present in the interventricular septum with 8 (2%) patients demonstrating NI‐LGE isolated to the left ventricular free wall. The presence of septal NI‐LGE was associated with increased risk of the primary endpoint on univariate analysis (HR 1.56, 95% CI 1.01–2.42, *p* = 0.04) (*Figure* *2B*) but not after adjustment for covariates (online supplementary *Table* *S11*). Neither extent nor pattern of NI‐LGE were associated with the primary endpoint on univariate or multivariate analysis (*Figure* *2C*,*D* and online supplementary *Table* *S11*). A summary of the univariable and multivariable analyses for the primary endpoint are detailed in online supplementary *Tables* *S12*–*S14*. Extended analysis is detailed in the online supplementary Results.

## Discussion

In this paper, we explore whether rare variants in DCM‐associated genes and non‐ischaemic patterns of myocardial scar are associated with adverse remodelling and outcomes in patients with CAD (*Graphical Abstract*). Such association may explain the broad variation in left ventricular dimensions and function for equivalent levels of infarct scar seen in this population. Our results support the notion that genetic variation contributes to left ventricular dysfunction and clinical outcomes in CAD. We additionally report that NI‐LGE is a marker of adverse remodelling in CAD but was not independently associated with cardiovascular outcomes and had no rare genetic basis.

### The impact of rare variants in dilated cardiomyopathy‐associated genes on phenotype and clinical outcomes in individuals with coronary artery disease

There is an increasing body of work describing a ‘two hit’ phenomena where environmental insults modulate expression of an underlying genetic susceptibility to cardiomyopathy; notably in peripartum cardiomyopathy and chemotherapy‐associated cardiomyopathy where an association between *TTN*tv and lower LVEF has previously been demonstrated.^3^, ^4^ Whether *TTN*tv impact cardiac phenotype in individuals with CAD is less clear. This has the potential for clinical utility in patients where there is an apparent discrepancy between the extent of myocardial infarction or CAD and the degree of ventricular remodelling. Our study demonstrates that, albeit infrequently detected, rare pathogenic variants in DCM‐associated genes appear to be associated with impaired biventricular systolic function in CAD, the result driven by *TTN*tv. This suggests that *TTN*tv can modify cardiac morphology in ischaemic heart disease, already the largest cause of left ventricular systolic dysfunction globally, and builds on prior data highlighting the broader role of titin in HF.^5^ We also demonstrate that rare pathogenic variants in DCM‐associated genes predict adverse outcome in individuals with CAD, which may inform risk stratification. These novel results align with increasing data detailing the prognostic relevance of rare variants across not only DCM cohorts,^17^, ^18^, ^19^ but also in individuals without phenotypic evidence of cardiomyopathy.^15^

### Non‐infarct pattern late gadolinium enhancement in patients with coronary artery disease

One key objective of our study was to explore the possibility that there is a rare genetic basis for non‐ischaemic myocardial scar in patients with stable CAD; a putative explanation that has been offered in prior commentary.^20^ Distinguishing cause from effect regarding the aetiology of NI‐LGE in patients with CAD is, however, challenging to definitively resolve. The association between the presence of NI‐LGE and greater left ventricular systolic dysfunction aligns with findings from DCM cohorts.^21^ Progressive ventricular dilatation is presumed to be an important driver of extracellular remodelling in these patients with the resulting increased wall stress activating key fibrogenic networks including the renin–angiotensin–aldosterone system, adrenergic stimuli, inflammatory cascades and redox signalling.^22^, ^23^ In patients with extensive CAD and adverse left ventricular remodelling, the increase in wall stress could theoretically promote similar LGE patterns as seen in non‐ischaemic pathologies. However, an alternative explanation is that patients with left ventricular dilatation in the context of limited CAD or myocardial infarction burden may have a primary cardiomyopathic process. It is important to consider that both CAD and DCM are common conditions, with an estimated population prevalence of approximately 1:60 and 1:250, respectively,^24^, ^25^ and on this basis and that of their independent pathophysiological drivers, it is entirely biologically plausible that the co‐existence of both pathologies may occur in some individuals. Our results suggest that rare protein altering variants in genes associated with DCM (including *TTN*tv) are uncommon in CAD patients with NI‐LGE and therefore not a predominant driver of this imaging biomarker. Importantly however, the greater left ventricular dilatation and lower LVEF in patients with NI‐LGE could not be explained by more severe CAD extent or a greater burden of myocardial infarction, raising the possibility of a second intercurrent pathological process. Future research assessing for a polygenic basis for NI‐LGE would be of value.

The results also demonstrate that NI‐LGE is not a strong independent predictor of outcomes in patients with CAD; data potentially suggesting that NI‐LGE is simply a marker of adverse remodelling in patients with CAD and that the risk of adverse clinical events associated with the HF syndrome is better captured by other variables. These results, however, deviate from prior studies that report an independent association between non‐infarct patterns of myocardial scar and adverse clinical outcomes in patients with CAD.^7^, ^20^ The divergence of these results from ours may hinge on the longer follow‐up period and difference in LVEF between groups in the current study, alongside our broader cardiovascular endpoint beyond solely arrhythmic events.

### Limitations

There are a limited number of participants with CAD in the UKB CMR study who have a rare pathogenic variant in a DCM‐associated gene (UKB Group 2, *n* = 8). The association between cardiac phenotype and rare variants in DCM‐associated genes should therefore be interpreted with caution. Additionally, population‐based cohorts do not permit the same degree of detail or accuracy of ascertainment of specific clinical features compared to a clinically recruited cohort. This therefore limits the ability to perform reliable multivariable outcome analysis. The London cohort is derived from a single tertiary centre and thus the generalizability of the results may be limited and selection bias cannot be excluded. However, the patient cohort represents a real‐world dataset of typical patients with stable CAD, referred from local cardiology clinics and a broad network of hospitals. Additionally, despite robust inclusion criteria for CAD in the London cohort, there is the potential for referral bias for patients with diagnostic uncertainty (e.g. higher suspicion of DCM). Importantly however, 80% of the patients in the London cohort were referred for ischaemia/viability testing with only 9% undergoing CMR for diagnostic uncertainty. The London cohort was predominantly male with only one female demonstrating NI‐LGE. This limits the generalizability of the results for female patients but aligns with data from prior DCM studies suggesting an increased prevalence of non‐ischaemic fibrosis in men as compared to females.^26^ Albeit that we found no significant difference in the burden of rare variants in DCM‐associated genes in patients with and without NI‐LGE, the sample size is small and larger multicentre studies would be of value. Furthermore, studies assessing for clusters of functional gene groups beyond *TTN* in patients with CAD, including evaluation of the prognostic role of arrhythmogenic DCM genes, would be of interest. Finally, our study only uses CMR undertaken at a single timepoint; future studies using serial cardiac imaging to assess dynamic left ventricular remodelling in patients with CAD and rare variants in DCM‐associated genes are needed.

## Conclusions

A small proportion of patients with stable CAD harbour rare pathogenic variants in DCM‐associated genes; our findings indicate that these variants may modulate left ventricular remodelling and increase the risk of adverse clinical outcomes in CAD. Genetic testing could be considered in patients with CAD and disproportionate left ventricular systolic dysfunction and may highlight a subgroup of individuals that would benefit from enhanced medical surveillance. NI‐LGE is similarly associated with adverse cardiac remodelling but, by contrast, is not an independent predictor of clinical outcomes in patients with stable CAD and prevalent infarct pattern scar. NI‐LGE was not found to have a rare genetic basis in this study and is likely to be primarily driven by increased wall stress in the setting of progressive systolic dysfunction.

## Supporting information


**Appendix S1.** Supporting Information.
