# Supplementary material for: Assessing the association between genetic and phenotypic features of dilated cardiomyopathy and outcome in patients with coronary artery disease
Source: Eur J Heart Fail. 2023 Oct 5;26(1):46–55. doi: 10.1002/ejhf.3033 (PMC11216513; doi:10.1002/ejhf.3033)
Supplement: Supplementary file 1 — Appendix S1. Supporting Information. [file EJHF-26-46-s001.docx]

**Supplemental materials online**

Contents page

[Supplementary Methods 2](#_Toc131710068)

[UK Biobank cohort 2](#_Toc131710069)

[Assessment for rare pathogenic variants in DCM-associated genes 2](#_Toc131710070)

[Clinical endpoints 4](#_Toc131710071)

[London cohort 5](#_Toc131710072)

[Assessment of imaging traits by Cardiovascular Magnetic Resonance 5](#_Toc131710073)

[DNA extraction and sequencing 5](#_Toc131710074)

[Clinical endpoints and follow up data collection 6](#_Toc131710075)

[Extended detail regarding the statistical analysis. 7](#_Toc131710076)

[Supplementary Results 8](#_Toc131710077)

[Extended analyses in the London Cohort 8](#_Toc131710078)

[Extended genetic analysis in the London Cohort 8](#_Toc131710079)

[Supplemental tables 9](#_Toc131710080)

[Supplementary figures 20](#_Toc131710081)

[Supplementary materials references 25](#_Toc131710082)

# Supplementary Methods

## UK Biobank cohort

The current study was conducted under Application Numbers 47602 and 40616. The UKB fields used to determine the diagnosis of CAD are detailed in supplementary table 1.

### Assessment for rare pathogenic variants in DCM-associated genes

Variants 100 bp +/- the region of 19 DCM-associated genes with definitive, strong, or moderate evidence for disease, were extracted from the whole exome sequencing data of 454,756 UKB participants to identify non-carriers. MANE, protein-altering variants that had a minor allele frequency (MAF) <0.1% in gnomAD and UKB were identified. Intron variants that were pathogenic in ClinVar were manually curated for functional evidence of splicing. Splice region variants and other splice variants (excluding essential splice and splice donor 5th base which were already included), were included if they were predicted to cause splicing through SpliceAI (threshold >0.8). Only TTN-tvs in PSI>90% exons or variants with a splice prediction or curation of pathogenic (with assessment) were included.

Steps:

1. 105,507 carriers of the 12,201 variants remaining include a list of individuals that are used to identify genotype negative individuals in the UKB. This includes 90,481 heterozygotes, 14,904 compound heterozygotes, and 119 homozygotes and 3 compound homozygotes (46 and 1 of which are also heterozygous for other variants, respectively). There are 349,249 genotype negatives.
2. The variant list was then shortened to only include the 12 definitive or strong evidence DCM genes (*BAG3, DES, DSP, FLNC, LMNA, MYH7, PLN, RBM20, SCN5A, TNNC1, TNNT2, TTN*). LOFTEE was used to identify low confidence predicted loss-of-function (pLoF) variants and identify protein altering variants (PAVs) that are mislabelled (e.g. NAGNAG sites). All predicted pLoF variants were annotated for prediction of nonsense-mediated decay (NMD) escaping; using the NMD plugin, a threshold of 55bp into the penultimate exon of the genes, and additional curation of frameshift variants upstream to the 55bp threshold but where the PTC is predicted to be within the threshold using HGVSp. Additionally, "missense_variant,splice_region_variant"s were only flagged as NMD escaping if they also had a prediction of splicing by SpliceAI. The variants were then filtered for disease-causing mechanisms; all *BAG3, LMNA, PLN, SCN5A, RBM20, and DSP*, PAVs were kept; only variants influencing gene product structure (indels, missense, start and stop lost) or gene product level but NMD escaping, were kept for *MYH7, DES, TNNC1, and TNNT2*; only variants predicted to influencing gene product level (e.g. frameshift, splice, stop gained) were kept for *TTN* and *FLNC*.
3. Carriers of the 7,549 variants remaining include a list of individuals that carry a DCM indeterminant variant. After exclusion for hypertrophic cardiomyopathy (HCM) variants (see Step3), 62,292 carriers were identified for the 7,467 variants, of which 56,815 were heterozygotes, 5,404 were compound heterozygotes, 72 were homozygotes (19 of which were also heterozygous for other variants) and 1 compound homozygote.
4. The variant list was then shortened to only include variants that met a filtering allele frequency (faf) of <0.00004 in gnomAD popmax faf [1] and as a MAF in UKB. Additionally, compound heterozyogous carriers of common *TTN*tvs (same two *TTN* variants identified in >10 individuals) were removed from the analysis as these were likely rescued.
5. Carriers of the 6,950 variants remaining include a list of individuals that carry a DCM indeterminant variant that is rare enough to have a substantial effect on disease. After exclusion for HCM variants (see below), 26,181 carriers were identified for the 6,868 variants, of which 25,207 were heterozygotes, 957 were compound heterozygotes, and 17 were homozygotes (1 of which was also heterozygous for another variant).
6. The variant list was then shortened to only include variants that would be called pathogenic/likely pathogenic if identified in a patient with DCM; using CardioClassifier [2] and ClinVar, the variants were manually curated if they had any evidence of pathogenicity. 1,351 variants were assessed, of which 1,018 had strong evidence of pathogenicity in DCM and 82 were identified with evidence of HCM. These 82 were excluded from Step2 onwards. Carriers of the 1,018 variants remaining include a list of individuals that carry a DCM pathogenic/likely pathogenic variant. 2,195 carriers were identified for the 1,018 variants, of which 2,181 were heterozygotes, 14 were compound heterozygotes, and there were no homozygotes.

The UKB whole-exome sequencing data was processed using Ensembl Variant Effect Predictor (VEP; version 105 [3]) with plugins for Genome Aggregation database (gnomAD) [4], LOFTEE [4] and SpliceAI [5]

### Clinical endpoints

For survival analysis, first occurrence outcomes data was used. In brief, UKB mapped: i) primary care data; ii) ICD-9 and ICD-10 codes from hospital inpatient data; iii) ICD-10 codes from Death Register records and; iv) self-reported medical conditions to ICD-10 codes. The earliest occurrence of each event in a participant’s lifetime was reported. The primary outcome for this study was a composite of all-cause mortality, cardiac arrest, or heart failure event (left ventricular assist device implantation [LVAD], cardiac transplantation). The secondary outcomes were i) all-cause mortality; ii) AF and iii) heart failure. For the non-fatal secondary outcomes, competing risk analysis was performed to account for death.

## London cohort

### Assessment of imaging traits by Cardiovascular Magnetic Resonance

CMR was undertaken at 1.5T (Siemens Aera). In brief, balanced steady-state free precession sequences were performed to acquire long and short-axis cine images. Gadopentetate dimeglumine or gadobutrol (0.1mmol/Kg) was injected intravenously and an inversion recovery gradient echo sequence was undertaken to obtain the LGE dataset as described previously [6]. Ventricular volumes and LV mass were calculated using a dedicated platform (CMRtools, Cardiovascular Imaging Solutions, London, United Kingdom) as previously described [7]. LGE quantification of ischaemic and non-ischaemic pattern fibrosis was performed by an independent Level 3 CMR operator using the full-width at half maximum method on specialised software (CVI42, Circle Cardiovascular Imaging Inc, Calgary, Canada).

NI-LGE was confirmed if present in: i) long- and short-axis planes; ii) 2 phase-encoding directions; and iii) extending beyond the localized ventricular insertion points and left ventricular outflow tract. NI-LGE was defined as linear mid-wall, subepicardial or in multiple patterns. The location of NI-LGE was categorised as ventricular septum, LV free wall or in both locations.

### DNA extraction and sequencing

DNA extraction was performed on available whole blood (273/453 cases) using automated platforms followed by targeted sequencing on the Solid 5500xl or Illumina NextSeq platforms. Samples from both platforms were jointly analysed, annotated and filtered according to a customised bioinformatics pipeline. Additionally, sequencing data from a healthy volunteer cohort and a reference population (Genome Aggregation database [gnomAD]) were used as additional comparator groups [4]. In brief, NGS reads underwent quality control per sample before being aligned against a reference human genome (hg19) using BWA (v0.7.17). For the Illumina platform any reads aligned with low mapping quality (Q<8) were filtered out. Aligned reads were pre-processed to identify any duplicates and recalibrate quality scores using the genome analysis toolkit (GATK v4) [8]. The GATK HaplotypeCaller was used for variant calling, and genomic vcf files were used for joint-genotyping following the GATK best practices [9]. Stringent hard-filtering was performed to all SNP and INDEL variants following GATK recommendations for germline short variant discovery. The Ensembl Variant Effects Predictor (VEP v104) was used for annotation of genomic consequence [10]. The annotated multi-sample VCF was taken forward for quality control and burden testing analysis. A secondary analysis using CardioClassifier [2] was performed as an additional annotation, but not filtering, step. *TTN* variants were only considered if they reside in constitutively expressed exons with PSI >0.9 [11].^.^ Rare variants in CAD cases and healthy controls were defined with overall minor allele frequency (MAF) <0.0001 in the highest disease-specific maximum credible population in gnomAD exomes (v2.1) (Popmax filtering AF (95% CI)) [1]. Alongside the primary genetic analysis, we also performed exploratory analysis testing the burden of TTNtv in patients with LVEF<35% versus i) LVEF ≥ 35%, ii) HVOLs and iii) the reference population gnomAD. Supplementary Table 1 details the curated genes and Supplementary Table 4 lists all detected rare variants.

### Clinical endpoints and follow up data collection

Patients were followed up using serial health questionnaires alongside general practitioner and hospital documentation. ICD reports, death certificates and post-mortem results were retrieved where necessary. The length of follow-up was defined from the date of CMR prior to consent until an endpoint was met or until the most recent contact date. Event times were determined from the date of the preceding CMR date for ≤10 years follow-up. Clinical outcomes were adjudicated by a panel of experienced cardiologists blinded to the CMR data. The primary endpoint was composite of all-cause mortality, life threatening arrhythmia and heart failure. Life threatening arrhythmia was defined as either a i) sudden cardiac death (death that occur unexpectedly, including situations where symptom duration was ≤1hr), following a confirmed arrhythmia/unsuccessful resuscitation or in circumstances where the patient was witnessed alive ≤24hr prior to death and no another identifiable cause of death was noted [12]; ii) major arrhythmic event defined as appropriate ICD shock for a ventricular tachyarrhythmia, successful resuscitation following ventricular fibrillation or haemodynamically unstable ventricular tachycardia [13]. A heart failure (HF) event was defined as i) HF hospitalisation (admission to hospital of ≥24hr/encompassing 1 calendar day with the initiation or optimisation of HF therapies) ii) HF death (death in the in the context of worsening clinical features of HF) or iii) cardiac transplantation or LVAD implantation.

### Extended detail regarding the statistical analysis.

Continuous variables were compared with Student t test or Mann-Whitney U tests based on normality of data. Categorical variables were compared with χ2 test or the Fisher exact test where applicable. Sensitivity analyses were conducted to: 1) assess the association between cardiac phenotype and NI-LGE in the patients with confirmed CAD (i.e. removing patients without documented CAD but a clinical history of myocardial infarction and ischaemic pattern LGE on CMR); 2) assess the association between cardiac phenotype and NI-LGE in patients with CAD, excluding patients with prior MI; 3) assess the association between cardiac phenotype and NI-LGE in patients with CAD, excluding patients with prior coronary revascularisation; 4) assess the association between cardiac phenotype and NI-LGE in patients with CAD, restricting the analysis to patients referred for CMR for ischaemia/viability testing.

# Supplementary Results

## Extended analyses in the London Cohort

Overall, 363 (80%) patients were referred for assessment of myocardial ischaemia/viability with 43 (9%) patients undergoing CMR for diagnostic uncertainty. Additional indications for CMR included evaluation of valvular disease, assessment for myocardial fibrosis or prior to cardiac device implantation. The proportion of patients referred for ischaemia/viability assessment was similar in those with and without NI-LGE (47/63 [74.6%] versus 316/390 [81.0%] respectively, p=0.24). The median number of days between CMR and recruitment was 0 days (IQR: 0-0 days). During a median follow up of 6.4 years (IQR: 5.1-7.9 years), 111 (25%) patients underwent PCI. Sensitivity analyses are detailed in Supplementary Tables 6-9.

## Extended genetic analysis in the London Cohort

There was no difference in age (p=0.17), sex distribution (p=0.62), proportion with prior myocardial infarction (p=0.28) or the presence of significant CAD (p=0.81) in patients with and without *TTN*tv (Supplementary Table 5). The burden of *TTN*-tv in patients with and without a LVEF<35% was 2.7% vs 0.6%, odds ratio 4.5, 95% CI 0.4-240.0, P=0.3. The burden of *TTN*-tv in patients with LVEF <35% compared to HVOLs was 2.7% vs 0.7%, odds ratio 3.9, 95% CI 0.7-15.1, P=0.06 respectively. There was increased burden of *TTN*-tv in patients with LVEF <35% as compared to the reference population gnomAD (1.4% vs 0.3%, odds ratio 4.6, 95% CI 0.9-13.6, P=0.03 respectively). The median infarct pattern mass of patients with and without a *TTN*tv was 8.6g [IQR 0.0-20.9g] vs 21.9g [IQR 9.9-35.1g] respectively, P=0.09

There was no significant difference in the burden of rare synonymous DCM variants between: i) CAD patients with and without NI-LGE (9.5% vs 13.8%, odds ratio 0.7, 95% CI 0.2-1.7, P=0.5); ii) CAD patients with and without LVEF <35% (14.5% vs 11.7%, odds ratio 1.3, 95% CI 0.6-2.8, P=0.6); iii) CAD patients with LVEF <35% and HVOLs (14.5% vs 13.9%, odds ratio 1.1, 95% CI 0.6-1.8, P=0.9); iv) CAD patients with LVEF <35% and the reference population gnomAD (7.7% vs 5.9%, odds ratio 1.3, 95% CI 0.8-2.2, P=0.3).

# Supplemental tables

| **Supplementary Table 1: CAD definitions used in UKB Groups** | |
| --- | --- |
| Self-reported | “heart attack/myocardial infarction”, “coronary artery bypass grafts (CABG)”, “triple heart bypass”, “coronary angioplasty (PTCA) +/- stent” |
| ICD-10 codes | I210 (Acute transmural myocardial infarction of anterior wall), I211 (Acute transmural myocardial infarction of inferior wall), I212 (Acute transmural myocardial infarction of other sites), I213 (Acute transmural myocardial infarction of unspecified site), I214 (Acute subendocardial myocardial infarction), I219 (Acute myocardial infarction, unspecified), I220 (Subsequent ST elevation (STEMI) myocardial infarction of anterior wall), I221 (Subsequent ST elevation (STEMI) myocardial infarction of inferior wall), I228 (Subsequent ST elevation (STEMI) myocardial infarction of other sites), I229 (Subsequent ST elevation (STEMI) myocardial infarction of unspecified site), I252 (Old myocardial infarction) |
| ICD-9 codes | 410 (Acute myocardial infarction), 411 (Other acute and subacute forms of ischaemic heart disease), 412 (Old myocardial infarction) |
| OPCS4 codes | K483 (Open angioplasty of coronary artery), K491 (Percutaneous transluminal balloon angioplasty of one coronary artery), K492 (Percutaneous transluminal balloon angioplasty of multiple coronary arteries), K493 (Percutaneous transluminal balloon angioplasty of bypass graft of coronary artery), K494 (Percutaneous transluminal cutting balloon angioplasty of coronary artery), K498 (Other specified transluminal balloon angioplasty of coronary artery), K499 (Unspecified transluminal balloon angioplasty of coronary artery), K751 (Percutaneous transluminal balloon angioplasty and insertion of 1-2 drug-eluting stents into coronary artery), K752 (Percutaneous transluminal balloon angioplasty and insertion of 3 or more drug-eluting stents into coronary artery), K753 (Percutaneous transluminal balloon angioplasty and insertion of 1-2 stents into coronary artery), K754 (Percutaneous transluminal balloon angioplasty and insertion of 3 or more stents into coronary artery NEC), K758 (Other specified percutaneous transluminal balloon angioplasty and insertion of stent into coronary artery), K759 (Unspecified percutaneous transluminal balloon angioplasty and insertion of stent into coronary artery, K401 (Saphenous vein graft replacement of one coronary artery), K402 (Saphenous vein graft replacement of two coronary arteries), K403 (Saphenous vein graft replacement of three coronary arteries), K404 (Saphenous vein graft replacement of four or more coronary arteries), K408 (Other specified saphenous vein graft replacement of coronary artery), K409 (Unspecified saphenous vein graft replacement of coronary artery), K411 (Autograft replacement of one coronary artery NEC), K412 (Autograft replacement of two coronary arteries NEC), K413 (Autograft replacement of three coronary arteries NEC), K414 (Autograft replacement of four or more coronary arteries NEC), K418 (Other specified other autograft replacement of coronary artery), K419 (Unspecified other autograft replacement of coronary artery), K421 (Allograft replacement of one coronary artery), K422 (Allograft replacement of two coronary arteries), K423 (Allograft replacement of three coronary arteries), K424 (Allograft replacement of four or more coronary arteries), K428 (Other specified allograft replacement of coronary artery), K429 (Unspecified allograft replacement of coronary artery), K431 (Prosthetic replacement of one coronary artery), K432 (Prosthetic replacement of two coronary arteries), K433 (Prosthetic replacement of three coronary arteries), K434 (Prosthetic replacement of four or more coronary arteries), K438 (Other specified prosthetic replacement of coronary artery), K439 (Unspecified prosthetic replacement of coronary artery), K441 (Replacement of coronary arteries using multiple methods), K442 (Revision of replacement of coronary artery), K448 (Other specified other replacement of coronary artery), K449 (Unspecified other replacement of coronary artery), K451 (Double anastomosis of mammary arteries to coronary arteries), K452 (Double anastomosis of thoracic arteries to coronary arteries NEC), K453 (Anastomosis of mammary artery to left anterior descending coronary artery), K454 (Anastomosis of mammary artery to coronary artery NEC), K455 (Anastomosis of thoracic artery to coronary artery NEC), K456 (Revision of connection of thoracic artery to coronary artery), K458 (Other specified connection of thoracic artery to coronary artery), K459 (Unspecified connection of thoracic artery to coronary artery) |

| **Supplementary Table 2: Genes assessed in the London Cohort** | |
| --- | --- |
| **Gene symbol** | **Variant class** |
| *DES* | TV + nTV |
| *DSP* | TV + nTV |
| *LMNA* | TV + nTV |
| *PLN* | TV + nTV |
| *RBM20* | TV + nTV |
| *SCN5A* | TV + nTV |
| *BAG3* | TV |
| *TTN* | TV* |
| *MYH7* | nTV |
| *TNNC1* | nTV |
| *TNNT2* | nTV |
| DCM genes used in the London cohort analysis. The variant classes are also listed. *TTN truncating variants were only included if identified in constitutively expressed exons with PSI<0.9. nTV = non-truncating; PSI = percentage spliced in; TV = truncating | |

| **Supplementary Table 3. Summary baseline characteristics from the UKB and London Cohorts** | | |
| --- | --- | --- |
| **Variable** | **UKB (n=31,957)** | **London cohort (n=453)** |
| Age, years | 73.4 (6.6) | 64.4 (9.9) |
| BMI, kg/m^2^ | 28.8 (4.8) | 27.8 (5.0) |
| Male | 23748 (74.3) | 390 (86.1) |
| Hypertension | 23699 (74.2) | 239 (52.8) |
| Diabetes mellitus | 8367 (26.2) | 130 (28.7) |
| LVEF, % | 59.5 (6.2)* | 46.9 (16.6) |
| BMI = body mass index; LVEF = left ventricular ejection fraction; UKB = UK biobank. Continuous variables are reported as mean (standard deviation) and categorical variables are reported as N (%). *LVEF measurements available for 1,600 participants in UKB (1,592 without pathogenic variants, 8 with pathogenic variants). | | |

| **Supplementary Table 4. Demographics of UK Biobank participants with coronary artery disease, stratified by pathogenic variants** | | | |
| --- | --- | --- | --- |
|  | **Pathogenic variants** | | |
| **Variable** | **Absent (N=31,774)** | **Present (N=183)** | **P-value** |
| **Age, years** | 74.0 (6.6) | 73.6 (6.1) | 0.38 |
| **Male** | 23,619 (74.3) | 129 (70.5) | 0.24 |
| **BMI, kg/m^2^** | 28.9 (4.8) | 28.5 (4.6) | 0.21 |
| **Hypertensive** | 23,564 (74.2) | 135 (73.8) | 0.93 |
| **Type 2 diabetes** | 8,322 (26.2) | 45 (24.6) | 0.68 |
| **LVEF, %*** | 56.7 (7.9) | 46.7 (10.3) | 0.0003 |
| LVEF = left ventricular ejection fraction  *LVEF measurements available for 1,600 participants in UKB (1,592 without pathogenic variants, 8 with pathogenic variants). Continuous variables are reported as mean (standard deviation) and categorical variables are reported as N (%) | | | |

| **Supplementary Table 5.** **All rare variants and protein consequences identified in the London cohort.** | | | |
| --- | --- | --- | --- |
| **Consequence** | **Gene** | **HGVSc** | **HGVSp** |
| missense_variant | DES | ENST00000373960.3:c.406C>G | ENSP00000363071.3:p.Leu136Val |
| missense_variant | SCN5A | ENST00000413689.1:c.5527G>C | ENSP00000410257.1:p.Val1843Leu |
| missense_variant | SCN5A | ENST00000413689.1:c.3835G>A | ENSP00000410257.1:p.Val1279Ile |
| missense_variant | SCN5A | ENST00000413689.1:c.3773G>C | ENSP00000410257.1:p.Trp1258Ser |
| missense_variant | SCN5A | ENST00000413689.1:c.3308C>T | ENSP00000410257.1:p.Ser1103Phe |
| missense_variant | DSP | ENST00000379802.3:c.484C>G | ENSP00000369129.3:p.Arg162Gly |
| missense_variant | DSP | ENST00000379802.3:c.1985A>G | ENSP00000369129.3:p.Asp662Gly |
| missense_variant | DSP | ENST00000379802.3:c.2552T>A | ENSP00000369129.3:p.Leu851Gln |
| missense_variant | DSP | ENST00000379802.3:c.3836C>T | ENSP00000369129.3:p.Ala1279Val |
| missense_variant | DSP | ENST00000379802.3:c.4915G>A | ENSP00000369129.3:p.Val1639Met |
| missense_variant | DSP | ENST00000379802.3:c.5363A>G | ENSP00000369129.3:p.Gln1788Arg |
| missense_variant | DSP | ENST00000379802.3:c.7622G>A | ENSP00000369129.3:p.Arg2541Lys |
| missense_variant | DSP | ENST00000379802.3:c.8513G>A | ENSP00000369129.3:p.Arg2838His |
| missense_variant | MYH7 | ENST00000355349.3:c.2682A>C | ENSP00000347507.3:p.Glu894Asp |
| missense_variant | MYH7 | ENST00000355349.3:c.1129G>A | ENSP00000347507.3:p.Gly377Ser |
| missense_variant | MYH7 | ENST00000355349.3:c.652G>T | ENSP00000347507.3:p.Asp218Tyr |
| frameshift_variant | TTN | ENST00000589042.1:c.79894del | ENSP00000467141.1:p.Glu26632AsnfsTer12 |
| stop_gained | TTN | ENST00000589042.1:c.59062G>T | ENSP00000467141.1:p.Glu19688Ter |
| frameshift_variant | TTN | ENST00000589042.1:c.45344del | ENSP00000467141.1:p.Val15115AspfsTer62 |
| stop_gained | TTN | ENST00000589042.1:c.7498C>T | ENSP00000467141.1:p.Gln2500Ter |

| **Supplementary Table 6.** **Demographics of patients in the London cohort, stratified by *TTN*tv** | | | |
| --- | --- | --- | --- |
|  | ***TTN*tv** | | |
| **Variable** | **Absent (N=269)** | **Present (N=4)** | **p-value** |
| **Age, years** | 65.1 (10.0) | 69.8 (5.3) | 0.17 |
| **Male** | 238 (88.5) | 4 (100.0) | 0.62 |
| **Significant CAD, n** | 251 (93.3) | 4 (100.0) | 0.81 |
| **Prior MI** | 200 (74.3) | 2 (50.0)* | 0.28 |
| **LVEF, %** | 40.8 (15.8) | 33.0 (14.7) | 0.37 |
| **Infarct pattern LGE mass (g)** | 21.9 (9.9-35.1) | 8.6 (0.0-20.9) | 0.09 |
| **Non-infarct pattern LGE, n** | 61 (22.7) | 2 (50.0) | 0.23 |
| CAD = coronary artery disease; LGE = late gadolinium enhancement; LVEF = left ventricular ejection fraction; IQR = interquartile range; MI = myocardial infarction; *TTN*tv = titin truncating variant. *1 patient had a history of MI several years prior to recruitment with subsequent coronary artery bypass grafting; there was transmural LGE on CMR. 1 patient had non-ST elevation myocardial infarction 3 years prior to recruitment with subsequent percutaneous coronary intervention to the left anterior descending artery. Continuous variables are reported as mean (standard deviation) or median (IQR) and categorical variables are reported as N (%). | | | |

| **Supplementary Table 7. Cardiac phenotype of patients in the London Cohort stratified by NI-LGE presence, only including patients with confirmed CAD.** | | | |
| --- | --- | --- | --- |
|  | **Non-infarct LGE** | | **p-value** |
|  | **No (n=366)** | **Yes (n=60)** |  |
| **LVEF, %** | 48.4 (16.0) | 38.1 (18.0) | <0.001 |
| **LVESVi, ml/m2** | 49.7 (28.3-75.1) | 84.7 (44.1-117.2) | <0.001 |
| **LVEDVi, ml/m2** | 103.1 (38.0) | 131.0 (48.4) | <0.001 |
| CAD = coronary artery disease; LVEDVi = indexed left ventricular end-diastolic volume; LVESVi = indexed left ventricular end-systolic volume; LVEF = left ventricular ejection fraction; IQR = interquartile range; NI-LGE = non-infarct pattern late gadolinium enhancement. Continuous variables are reported as mean (standard deviation) or median (IQR) and categorical variables are reported as N (%). | | | |

| **Supplementary Table 8. Cardiac phenotype of patients in the London Cohort stratified by NI-LGE presence, excluding patients with prior myocardial infarction.** | | | |
| --- | --- | --- | --- |
|  | **Non-infarct LGE** | | **p-value** |
|  | **No (n=95)** | **Yes (n=28)** |  |
| **LVEF, %** | 54.0 (18.5) | 37.6 (20.2) | <0.001 |
| **LVESVi, ml/m2** | 29.4 (20.1-70.2) | 88.2 (32.0-126.4) | <0.001 |
| **LVEDVi, ml/m2** | 92.8 (38.0) | 136.1 (57.4) | <0.001 |
| CAD = coronary artery disease; LVEDVi = indexed left ventricular end-diastolic volume; LVESVi = indexed left ventricular end-systolic volume; LVEF = left ventricular ejection fraction; IQR = interquartile range; NI-LGE = non-infarct pattern late gadolinium enhancement. Continuous variables are reported as mean (standard deviation) or median (IQR) and categorical variables are reported as N (%). | | | |

| **Supplementary Table 9. Cardiac phenotype of patients in the London Cohort stratified by NI-LGE presence, excluding patients with prior coronary revascularisation.** | | | |
| --- | --- | --- | --- |
|  | **Non-infarct LGE** | | **p-value** |
|  | **No (n=125)** | **Yes (n=25)** |  |
| **LVEF, %** | 43.5 (15.2) | 36.8 (20.5) | 0.06 |
| **LVESVi, ml/m2** | 61.0 (37.1-90.5) | 88.8 (56.9-142.3) | 0.02 |
| **LVEDVi, ml/m2** | 110.9 (39.4) | 138.4 (55.3) | 0.004 |
| CAD = coronary artery disease; LVEDVi = indexed left ventricular end-diastolic volume; LVESVi = indexed left ventricular end-systolic volume; LVEF = left ventricular ejection fraction; IQR = interquartile range; NI-LGE = non-infarct pattern late gadolinium enhancement. Continuous variables are reported as mean (standard deviation) or median (IQR) and categorical variables are reported as N (%). | | | |

| **Supplementary Table 10. Cardiac phenotype of patients in the London Cohort stratified by NI-LGE presence, only including patients referred for ischaemia/viability testing.** | | | |
| --- | --- | --- | --- |
|  | **Non-infarct LGE** | | **p-value** |
|  | **No (n=316)** | **Yes (n=47)** |  |
| **LVEF, %** | 49.8 (15.9) | 41.1 (18.8) | <0.001 |
| **LVESVi, ml/m2)** | 47.3 (27.3-72.3) | 78.1 (34.8-108.1) | <0.001 |
| **LVEDVi, ml/m2** | 99.9 (36.4) | 123.1 (44.1) | <0.001 |
| CAD = coronary artery disease; LVEDVi = indexed left ventricular end-diastolic volume; LVESVi = indexed left ventricular end-systolic volume; LVEF = left ventricular ejection fraction; IQR = interquartile range; NI-LGE = non-infarct pattern late gadolinium enhancement. Continuous variables are reported as mean (standard deviation) or median (IQR) and categorical variables are reported as N (%). | | | |

| **Supplementary Table 11. Univariable and multivariable Cox regression analyses for the primary endpoint in the London cohort** | | | | | | |
| --- | --- | --- | --- | --- | --- | --- |
|  | **Unadjusted** | | **Adjusted Model 1^1^** | | **Adjusted Model 2^2^** | |
|  | **HR (95% CI)** | **p-value** | **HR (95% CI)** | **p-value** | **HR (95% CI)** | **p-value** |
| **Non-infarct LGE** |  |  |  |  |  |  |
| 0-2 years | 0.97 (0.46, 2.04) | 0.93 | 0.47 (0.21, 1.04) | 0.06 | 0.57 (0.26, 1.23) | 0.15 |
| 2-10 years | 1.45 (0.91, 2.29) | 0.11 | 1.01 (0.61, 1.66) | 0.97 | 0.91 (0.57, 1.48) | 0.72 |
| **Midwall non-infarct LGE** |  |  |  |  |  |  |
| 0-2 years | 1.10 (0.52, 2.33) | 0.80 | 0.50 (0.22, 1.12) | 0.09 | 0.60 (0.28, 1.31) | 0.20 |
| 2-10 years | 1.54 (0.96, 2.47) | 0.07 | 1.02 (0.61, 1.70) | 0.94 | 0.92 (0.57, 1.51) | 0.75 |
| **Septal non-infarct LGE** |  |  |  |  |  |  |
| 0-2 years | 1.18 (0.56, 2.49) | 0.67 | 0.50 (0.22, 1.13) | 0.10 | 0.61 (0.28, 1.33) | 0.21 |
| 2-10 years | 1.75 (1.09, 2.80) | 0.02 | 1.08 (0.64, 1.81) | 0.78 | 0.97 (0.59, 1.60) | 0.91 |
| **Extent of non-infarct LGE** |  |  |  |  |  |  |
| 0-2 years |  | 0.41 |  | 0.28 |  | 0.57 |
| <3.06% (vs 0%) | -- |  | -- |  | -- |  |
| ≥3.06% (vs 0%) | 1.87 (0.89, 3.94) |  | 0.82 (0.37, 1.80) |  | 1.08 (0.50, 2.32) |  |
| 2-10 years |  | 0.10 |  | 0.97 |  | 0.77 |
| <3.06% (vs 0%) | 1.33 (0.71, 2.47) |  | 1.02 (0.52, 1.99) |  | 0.89 (0.47, 1.68) |  |
| ≥3.06% (vs 0%) | 1.59 (0.85, 2.97) |  | 1.01 (0.53, 1.92) |  | 0.94 (0.50, 1.77) |  |
| Univariate and multivariable Cox regression analysis investigating the association between presence, pattern, location and extent of NI-LGE with the primary endpoint of all-cause mortality, life threatening arrhythmia or major heart failure event. The Kaplan-Meier plots cross at approximately 2 years and thus the regression analysis was separated for 0-2 years and 2-10 years. ^1^Model adjusted for a subset of variables in Table 1 identified in a forward stepwise procedure (LVEF, age, atrial fibrillation, diabetes, NYHA functional score, LVEDVi and history of prior myocardial infarction). ^2^Model adjusted for LVEF, age and sex.  CI = confidence interval; LGE = late gadolinium enhancement; HR = hazard ratio; LVEDVi = indexed left ventricular end-diastolic volume; LVEF = left ventricular ejection fraction; NI-LGE = non-infarct pattern LGE; NYHA = New York Heart Association. | | | | | | |

| **Supplementary Table 12. Univariable associations for the composite of all-cause death, major heart failure events and life-threatening arrhythmia.** | | |
| --- | --- | --- |
| **Variable** | **HR (95% CI)** | **P-value** |
| **Age (per 10 years)** | 1.38 (1.17, 1.63) | <0.001 |
| **Female** | 0.71 (0.45, 1.14) | 0.16 |
| **Caucasian** | 1.00 (0.69, 1.45) | 0.99 |
| **BMI (kg/m2)** | 1.01 (0.98, 1.04) | 0.43 |
| **Hypertension** | 1.09 (0.82, 1.47) | 0.55 |
| **Significant CAD** | 1.80 (0.74, 4.39) | 0.19 |
| **CAD type** |  | <0.001 |
| Single vessel | 0.40 (0.27, 0.60) |  |
| 2 vessels | 0.73 (0.52, 1.03) |  |
| 3 vessels | Reference group |  |
| **Diabetes** | 1.77 (1.31, 2.39) | <0.001 |
| **Hypercholesterolemia** | 0.91 (0.63, 1.31) | 0.60 |
| **Prior MI** | 1.74 (1.20, 2.53) | 0.004 |
| **History of PCI** | 0.69 (0.52, 0.93) | 0.01 |
| **History of CABG** | 1.93 (1.43, 2.59) | <0.001 |
| **Baseline atrial fibrillation** | 2.18 (1.57, 3.01) | <0.001 |
| **NYHA** |  | <0.001 |
| 1 | 0.61 (0.42, 0.87) |  |
| 2 | Reference group |  |
| 3 or 4 | 1.34 (0.94, 1.91) |  |
| **Diuretic** | 3.31 (2.41, 4.54) | <0.001 |
| **Beta-blocker** | 1.27 (0.88, 1.83) | 0.20 |
| **ACE-i/ARB** | 1.60 (1.01, 2.52) | 0.04 |
| **Lipid-lowering drug** | 0.82 (0.53, 1.28) | 0.39 |
| **Aldesterone antagonist** | 2.42 (1.79, 3.27) | <0.001 |
| **LVEF (%)** | 0.96 (0.95, 0.97) | <0.001 |
| **LV mass indexed (g/m2)** | 1.01 (1.01, 1.02) | <0.001 |
| **LVESVi (ml/m2)** | 2.69 (2.12, 3.41) | <0.001 |
| **LVEDVi (ml/m2)** | 4.51 (3.04, 6.68) | <0.001 |
| **RVEF (%)** | 0.98 (0.97, 0.99) | <0.001 |
| ACEi = angiotensin-converting enzyme inhibitor; ARB = angiotensin II receptor blocker; BMI = body mass index; CABG = coronary artery bypass grafting; CAD = coronary artery disease; CI = confidence interval; HR = hazard ratio; IQR = interquartile range; LGE = late gadolinium enhancement; LVEDVi = indexed left ventricular end-diastolic volume; LVEF = left ventricular ejection fraction; MI = myocardial infarction; NI-LGE = non-infarct pattern late gadolinium enhancement; NYHA = New York Heart Association; RVEDVi = indexed right ventricular end-systolic volume; RVEF = right ventricular ejection fraction; PCI = percutaneous coronary intervention. | | |

| **Supplementary Table 13. Full Adjusted Model 1 for the association between non-infarct LGE and the composite of all-cause death, major heart failure events and life-threatening arrhythmia.** | | | | |
| --- | --- | --- | --- | --- |
| **Adjusted Model 1** | **0-2 years** | | **2-5 years** | |
|  | **HR (95% CI)** | **P** | **HR (95% CI)** | **P** |
| Non-infarct LGE | 0.47 (0.21, 1.04) | 0.06 | 1.01 (0.61, 1.66) | 0.97 |
| LVEF (per 10%) | 0.71 (0.54, 0.94) | 0.02 | 0.87 (0.72, 1.05) | 0.16 |
| Atrial fibrillation | 1.21 (0.64, 2.28) | 0.57 | 1.63 (1.09, 2.43) | 0.02 |
| Diabetes | 1.99 (1.17, 3.38) | 0.02 | 1.65 (1.13, 2.39) | 0.009 |
| NYHA |  | 0.001 |  | 0.13 |
| 1 | Reference group |  | Reference group |  |
| 2 | 1.89 (0.87, 4.08 |  | 1.31 (0.86, 1.99) |  |
| 3 or 4 | 3.59 (1.64, 7.87) |  | 1.46 (0.86, 2.46) |  |
| LVEDVi (log) | 1.76 (0.56, 5.54) | 0.33 | 2.87 (1.23, 6.65) | 0.01 |
| Age (per 10 years) | 1.20 (0.90, 1.60) | 0.21 | 1.45 (1.18, 1.79) | <0.001 |
| Prior MI | 0.83 (0.45, 1.51) | 0.54 | 2.17 (1.30, 3.64) | 0.003 |
| CI = confidence interval; HR = hazard ratio; LGE = late gadolinium enhancement; LVEDVi = indexed left ventricular end-diastolic volume; LVEF = left ventricular ejection fraction; MI = myocardial infarction. | | | | |
|  |  |  |  |  |
|  |  |  |  |  |
|  |  |  |  |  |
|  |  |  |  |  |
|  |  |  |  |  |
| **Supplementary Table 14. Full Adjusted Model 2 for the association between non-infarct LGE and the composite of all-cause death, major heart failure events and life-threatening arrhythmia.** | | | | |
| **Adjusted Model 2** | **0-2 years** | | **2-5 years** | |
|  | **HR (95% CI)** | **P** | **HR (95% CI)** | **P** |
| Non-infarct LGE | 0.57 (0.26, 1.23) | 0.15 | 0.91 (0.57, 1.48) | 0.72 |
|  |  |  |  |  |
| LVEF (per 10%) | 0.62 (0.52, 0.75) | <0.001 | 0.70 (0.62, 0.78) | <0.001 |
| Age (per 10 years) | 1.19 (0.91, 1.56) | 0.21 | 1.49 (1.21, 1.83) | <0.001 |
| Female | 1.01 (0.46, 2.26) | 0.97 | 0.79 (0.44, 1.42) | 0.44 |
| CI = confidence interval; HR = hazard ratio; LGE = late gadolinium enhancement; LVEF = left ventricular ejection fraction. | | | | |

# Supplementary figures

| **A** |
| --- |
| 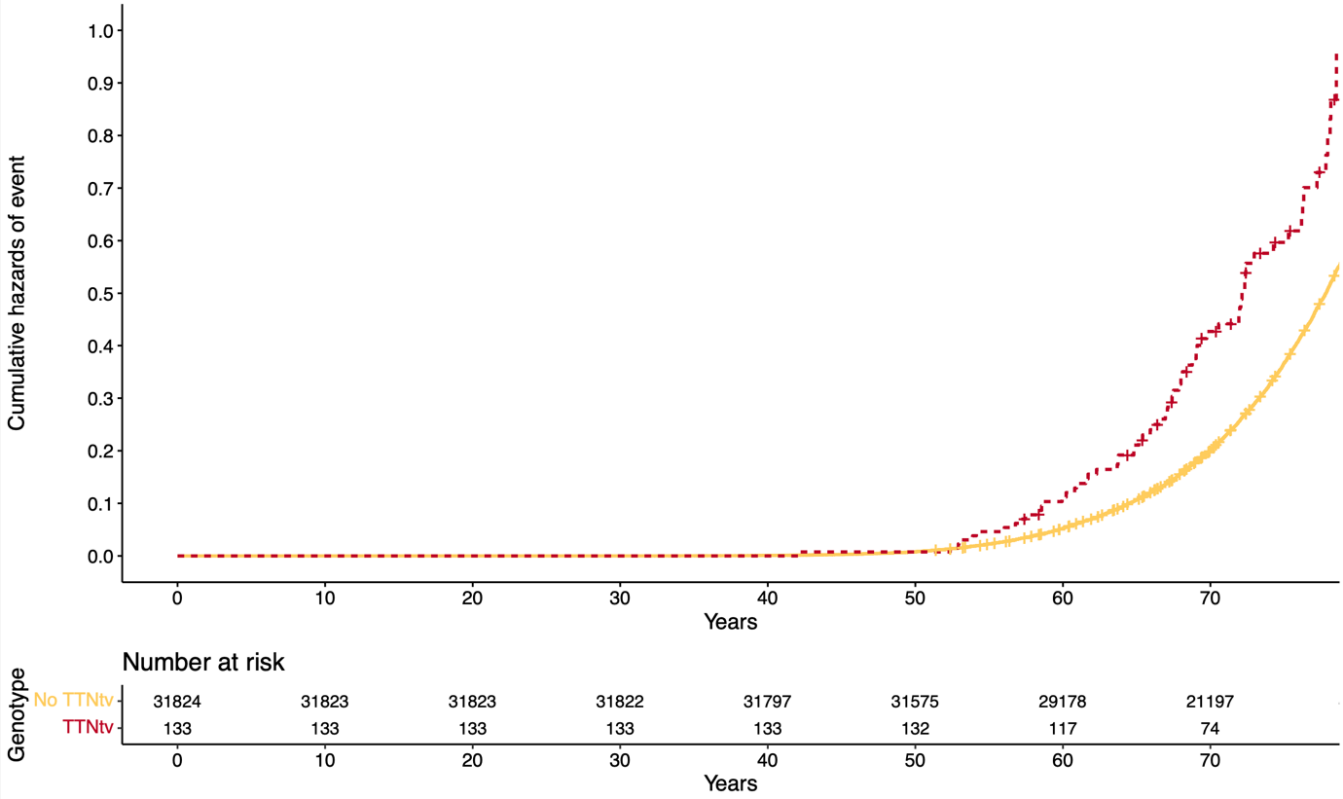 |
| B |
| 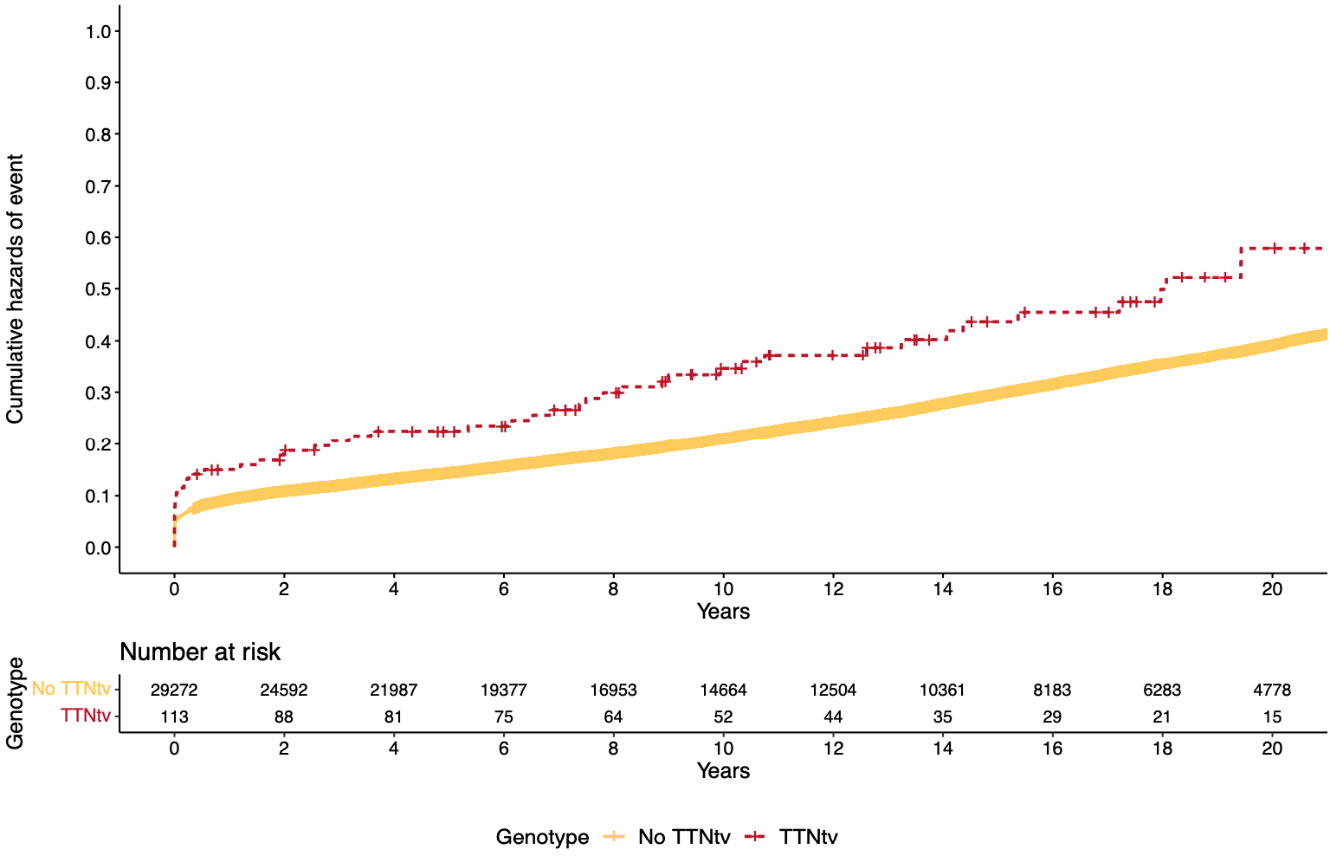 |
| Supplementary Figure 1. Clinical outcomes of UKB participants with coronary artery disease stratified by presence or absence of a *TTN*tv.  **A**: Plot demonstrating an increased lifetime risk of the primary endpoint in UKB individuals with CAD (N=31,957) and a *TTN*tv (N=133). **B**: Plot demonstrating cumulative hazard of incident primary outcome after diagnosis of CAD in UKB individuals (N=29,272) stratified by *TTN*tv status (N=113). Primary outcome includes all cause mortality, heart failure event (hospitalisation, LVAD implantation or transplant), and cardiac arrest. All models adjusted for age, age^2^, sex, and first ten genetic principal components. CAD = coronary artery disease; MACE = major adverse cardiovascular event; TTNtv = titin truncating variant; UKB = UK Biobank. |

|  |
| --- |
| Supplemental Figure 2: Derivation of the London cohort  Flow diagram describing the curation of the study cohort. ACS = acute coronary syndrome; CAD = coronary artery disease; CMR = cardiovascular magnetic resonance; ICD = implantable cardioverter defibrillator; LGE = late gadolinium enhancement; UK = United Kingdom. |

| 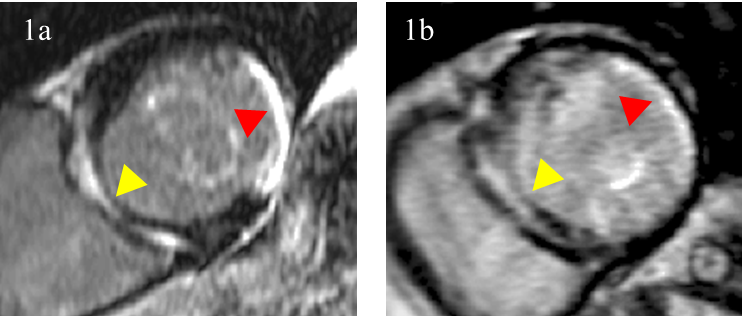 |
| --- |
| Supplementary Figure 3. Examples of NI-LGE in patients with known CAD.  LGE-CMR images from unique patients with CAD in the London cohort. 1a) Individual with previous MI and coronary revascularisation, LVEF 37% on CMR. Transmural infarction (red arrow) of the lateral wall and mid-wall fibrosis of the ventricular septum (yellow arrow); b) Individual with known three vessel disease and LVEF 36% on CMR. Subendocardial infarction (red arrow) of the lateral wall and mid-wall fibrosis of the ventricular septum (yellow arrow).  CMR = cardiovascular magnetic resonance; NI-LGE = non-infarct pattern late gadolinium enhancement; LVEF = left ventricular ejection fraction; MI = myocardial infarction. |

| 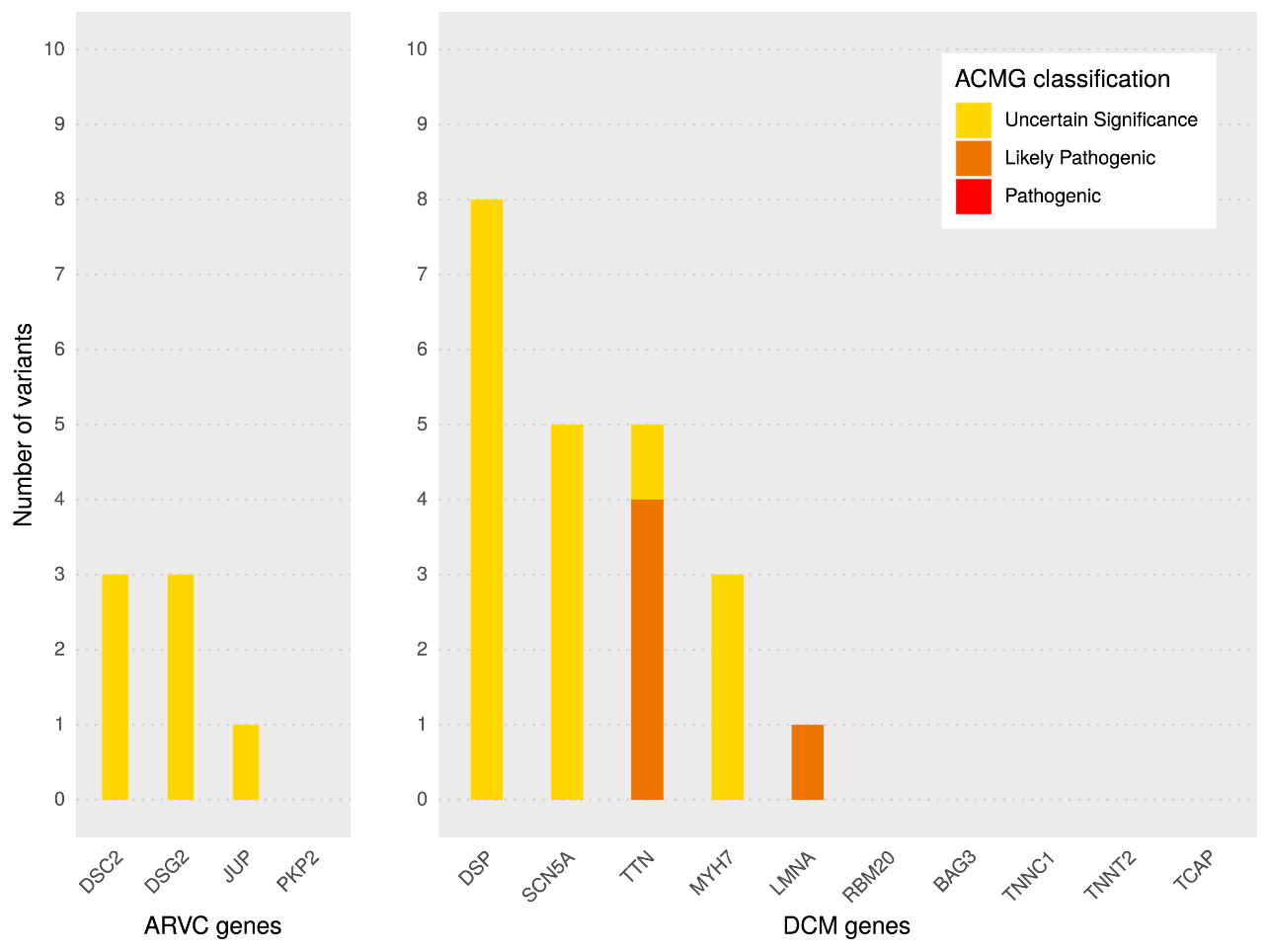 |
| --- |
| Supplementary Figure 4. CardioClassifier output in the London Cohort.  All protein-altering variants in ACM/ARVC and DCM genes observed in the London cohort assigned as variants of unknown significance or likely pathogenic based on ACMG criteria applied through the semi-automated computational decision-support tool, CardioClassifier.  ACM/ = arrhythmogenic cardiomyopathy/ arrhythmogenic right ventricular cardiomyopathy; DCM = dilated cardiomyopathy. |

# Supplementary materials references

[1] Whiffin N, Minikel E, Walsh R, O’Donnell-Luria AH, Karczewski K, Ing AY, et al. Using high-resolution variant frequencies to empower clinical genome interpretation. Genet Med 2017;19:1151–8. https://doi.org/10.1038/gim.2017.26.

[2] Whiffin N, Walsh R, Govind R, Edwards M, Ahmad M, Zhang X, et al. CardioClassifier: disease- and gene-specific computational decision support for clinical genome interpretation. Genet Med 2018;20:1246–54. https://doi.org/10.1038/gim.2017.258.

[3] McLaren W, Pritchard B, Rios D, Chen Y, Flicek P, Cunningham F. Deriving the consequences of genomic variants with the Ensembl API and SNP Effect Predictor. Bioinformatics 2010;26:2069–70. https://doi.org/10.1093/bioinformatics/btq330.

[4] Karczewski KJ, Francioli LC, Tiao G, Cummings BB, Alföldi J, Wang Q, et al. The mutational constraint spectrum quantified from variation in 141,456 humans. Nature 2020;581:434–43. https://doi.org/10.1038/s41586-020-2308-7.

[5] Jaganathan K, Kyriazopoulou Panagiotopoulou S, McRae JF, Darbandi SF, Knowles D, Li YI, et al. Predicting Splicing from Primary Sequence with Deep Learning. Cell 2019;176:535-548.e24. https://doi.org/10.1016/j.cell.2018.12.015.

[6] Assomull RG, Prasad SK, Lyne J, Smith G, Burman ED, Khan M, et al. Cardiovascular magnetic resonance, fibrosis, and prognosis in dilated cardiomyopathy. J Am Coll Cardiol 2006;48:1977–85. https://doi.org/10.1016/j.jacc.2006.07.049.

[7] Halliday BP, Gulati A, Ali A, Guha K, Newsome S, Arzanauskaite M, et al. Association Between Midwall Late Gadolinium Enhancement and Sudden Cardiac Death in Patients With Dilated Cardiomyopathy and Mild and Moderate Left Ventricular Systolic Dysfunction. Circulation 2017;135:2106–15. https://doi.org/10.1161/CIRCULATIONAHA.116.026910.

[8] van der Auwera, G. A; O’Connor BD. Genomics in the Cloud: Using Docker, GATK, and WDL in Terra. O’Reilly Media; 2020.

[9] van der Auwera GA, Carneiro MO, Hartl C, Poplin R, del Angel G, Levy-Moonshine A, et al. From FastQ data to high confidence variant calls: the Genome Analysis Toolkit best practices pipeline. Curr Protoc Bioinformatics 2013;43:11.10.1-11.10.33. https://doi.org/10.1002/0471250953.bi1110s43.

[10] McLaren W, Gil L, Hunt SE, Riat HS, Ritchie GRS, Thormann A, et al. The Ensembl Variant Effect Predictor. Genome Biol 2016;17:122. https://doi.org/10.1186/s13059-016-0974-4.

[11] Roberts AM, Ware JS, Herman DS, Schafer S, Baksi J, Bick AG, et al. Integrated allelic, transcriptional, and phenomic dissection of the cardiac effects of titin truncations in health and disease. Sci Transl Med 2015;7:270ra6. https://doi.org/10.1126/scitranslmed.3010134.

[12] Hicks KA, Tcheng JE, Bozkurt B, Chaitman BR, Cutlip DE, Farb A, et al. 2014 ACC/AHA Key Data Elements and Definitions for Cardiovascular Endpoint Events in Clinical Trials. Circulation 2015;132:302–61. https://doi.org/10.1161/CIR.0000000000000156.

[13] Buxton AE, Calkins H, Callans DJ, DiMarco JP, Fisher JD, Greene HL, et al. ACC/AHA/HRS 2006 key data elements and definitions for electrophysiological studies and procedures: a report of the American College of Cardiology/American Heart Association Task Force on Clinical Data Standards (ACC/AHA/HRS Writing Committee to Develop D. J Am Coll Cardiol 2006;48:2360–96. https://doi.org/10.1016/j.jacc.2006.09.020.
